# Constitutive Modeling of Rheological Behavior of Cement Paste Based on Material Composition

**DOI:** 10.3390/ma18132983

**Published:** 2025-06-24

**Authors:** Chunming Lian, Xiong Zhang, Lu Han, Wenbiao Lin, Weijun Wen

**Affiliations:** 1 Key Laboratory of Advanced Civil Engineering Materials of Education Ministry, School of Material Science and Technology, Tongji University, 4800 Cao’an Road, Shanghai 201804, China; zhangxiong@tongji.edu.cn; 2 China Construction Eighth Bureau Science and Technology Construction Co., Ltd., 899 Gaoke West Road, Shanghai 201804, China; whosname12@163.com (L.H.); linwenbiao@cscec.com (W.L.); wenweijun@cscec.com (W.W.)

**Keywords:** cement paste rheology, constitutive model, mineral admixtures, solid volume fraction

## Abstract

The rheological behavior of cementitious paste plays a pivotal role in determining the workability, pumpability, and uniformity of fresh concrete. Classical rheological models often struggle to capture the complex flocculation and hydration effects inherent in cement-based systems, and they typically depend on parameters that are difficult to measure directly, limiting their practical utility. This study presents a novel composition-based constitutive model that introduces a virtual maximum packing fraction ($\phi_{max}$) to account for interparticle flocculation and entrapped water effects. By establishing quantitative relationships between powder characteristics—such as particle size and specific surface area—and rheological parameters, the model enables physically interpretable and measurable predictions of yield stress and plastic viscosity. Our validation against 65 paste formulations with varying water-to-binder ratios, mineral admixture types and dosages, and superplasticizer contents demonstrates strong predictive accuracy (R^2^ > 0.98 for plain pastes and >0.85 for blended systems). The influence of superplasticizers is effectively captured through modifications to $\phi_{max}$, allowing the model to remain both robust and parameter efficient. This framework supports forward prediction of paste rheology from raw material properties, offering a valuable tool for intelligent mix design in high-performance concrete applications such as self-consolidating and 3D-printed concrete.

## 1. Introduction

The rheological behavior of cementitious pastes is paramount in dictating the workability, pumpability, and pre-hardening uniformity of fresh concrete [1]. An adequate volume of paste possessing suitable rheological properties is indispensable for ensuring the effective dispersion and lubrication of aggregate particles. Deficiencies in either paste volume or its flow characteristics can severely compromise the material, leading to critical issues such as aggregate interlocking, pumping blockages, and ultimately, poor casting performance [2,3].

Unlike inert suspensions, cement paste is a chemically reactive and multiscale system composed of water, fine powders smaller than 75 µm (including cement, supplementary cementitious materials, and micro-fillers), entrained air, and various chemical admixtures. Its flow behavior is governed by a complex interplay of interparticle forces, such as gravity-induced settling, van der Waals attraction, electrostatic repulsion, mechanical friction, and capillary effects [4,5]. During mixing, water initially fills the interstitial voids between particles, and any surplus water forms lubricating films that reduce friction. However, when water is insufficient, the probability of particle–particle contact and collision increases significantly, leading to a percolated network of interactions that profoundly resists flow. Consequently, flow only commences when the applied shear stress overcomes this internal resistance, clearly demonstrating the yield stress nature of cement paste [6].

The chemical reactivity of cement particles further complicates the rheological response. Upon hydration, cement particles develop surface charges which—depending on crystal face and reaction stage—can differ in polarity and magnitude. This dynamic electrochemical condition fosters the formation of flocculated structures through a combination of van der Waals attraction and insufficient repulsive barriers [7,8]. These flocs effectively entrap mixing water, consequently reducing the amount of free water available for lubrication and concurrently increasing both the plastic viscosity and the yield stress of the paste over time, a phenomenon particularly critical during mixing and transport [9]. Furthermore, these flocculated structures are sensitive to applied shear: they break down under high shear rates, leading to a shear-thinning response, which is commonly described as thixotropy [10]. The kinetics of floc breakdown and reformation significantly influence flow regime transitions and the overall pumpability of the mixture [11,12].

To overcome the limitations imposed by these flocculated structures, chemical admixtures—particularly polycarboxylate ether (PCE)-based superplasticizers—are commonly employed. These admixtures adsorb onto cement particles primarily through their hydrophobic main chains, while their hydrophilic side chains extend into the aqueous phase, generating both electrostatic repulsion and steric hindrance [13,14]. This sophisticated dual mechanism effectively facilitates the dispersion of particles and the breakdown of flocs, thereby releasing entrapped mixing water. Consequently, a dramatic enhancement in the paste’s rheological performance is observed, even at low water-to-binder ratios [15]. The efficacy of these admixtures in controlling rheology, including their impact on both yield stress and plastic viscosity, is crucial for optimizing flow characteristics across various shear rates, a concept also explored in studies on cement grouts [9].

The inclusion of supplementary cementitious materials (SCMs), such as fly ash, slag, and limestone powder, introduces additional complexities to the rheological system. Although often similar to cement in particle size distribution, SCMs exhibit distinct physicochemical properties—including variations in surface charge density, hydration reactivity, and affinity for admixtures—that significantly alter interparticle interactions and consequently modify rheological behavior [16,17,18]. These effects are frequently nonlinear and highly dependent on the specific type and dosage of SCMs incorporated [19]. Understanding these interactions is critical, as they can also influence the time-dependent characteristics and flow properties, like observations in other cement-based systems like grouts where water–cement ratio and material interactions dictate flow patterns and diffusion [20].

Despite numerous experimental and modeling efforts, predictive frameworks for cement paste rheology remain limited. Classical models, such as those proposed by Krieger–Dougherty, Bingham, and Herschel–Bulkley, while offering utility for simpler particulate suspensions, often fall short in complex cementitious systems. This inadequacy primarily stems from their inability to accurately capture critical phenomena like flocculation, particle hydration, and the evolution of surface properties over time [14,21,22]. Moreover, key model parameters—including the effective solid volume fraction and the maximum packing density—are inherently difficult to measure directly, as they are significantly influenced by evolving floc morphology and the content of adsorbed water. Furthermore, accurately predicting time-dependent behaviors and flow regime transitions, which are critical for practical applications like pumping and grouting, remains a significant challenge for these traditional models. Addressing these measurement complexities requires innovative approaches to defining effective volume concentrations.

To address these challenges, this study develops a constitutive rheological model specifically designed for cementitious pastes. The model incorporates measurable powder properties—such as particle size distribution and specific surface area—along with key compositional parameters including water-to-binder ratio, mineral admixture type and content, and superplasticizer dosage.

A central innovation of the model is the introduction of a virtual maximum packing fraction ($\phi_{max}$). This parameter represents the effective packing limit of the powder in the presence of water and admixtures. It reflects the influence of flocculated structures and entrapped water, which typically hinder the direct measurement of effective solid volume fraction. Unlike conventional models, this approach uses the absolute solid volume fraction (*ϕ*), easily calculated from powder density and water-to-binder ratio, as the base input.

By integrating $\phi_{max}$, the model enables simplified yet accurate prediction of yield stress and plastic viscosity, capturing their evolution over time. The model is calibrated and validated using a broad dataset that covers various mix designs and accounts for time-dependent behavior. It links microstructural features to macroscopic rheological response and enhances understanding of flow regime transitions.

This framework provides a practical and robust tool for designing high-performance cementitious materials. It advances beyond traditional empirical models and is applicable to a wide range of systems, including cement grouts and other cement-based composites.

## 2. Materials and Methods

### 2.1. Raw Materials

The binder system employed in this study consisted of an ordinary Portland cement (OPC) conforming to the Chinese standard GB 175-2007 [23] (P·O 42.5 grade, Conch brand). The physical properties of the cement are summarized in Table 1. The Blaine fineness was measured at 317 m^2^/kg, and the specific gravity was 2.99 g/cm^3^. Initial and final setting times were 193 and 266 min, respectively. The flexural and compressive strengths reached 9.3 MPa and 43.2 MPa at 28 days under standard curing conditions.

Three types of supplementary cementitious materials (SCMs) were used: Class F fly ash (FA) conforming to GB/T 1596-2017 [24], S95-grade ground granulated blast furnace slag (SL) in accordance with GB/T 18046-2008 [25], and ground limestone powder (ST) in accordance with GB/T 35164-2017 [26]. The bulk densities and Blaine surface areas of these powders, determined using Le Chatelier flask and laser diffraction methods, are listed in Table 2. To further account for the packing behavior in paste rheology modeling, the surface area per unit volume was also calculated.

**Figure 1:**
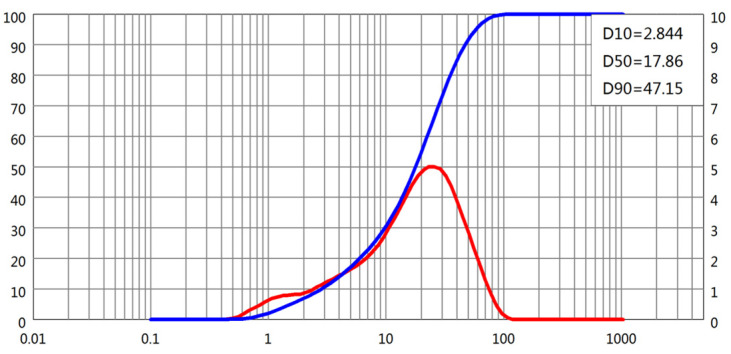
Particle size distribution of cement.

**Figure 2:**
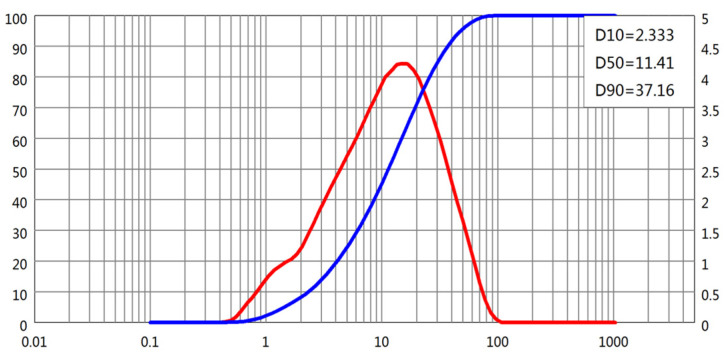
Particle size distribution of fly ash.

**Figure 3:**
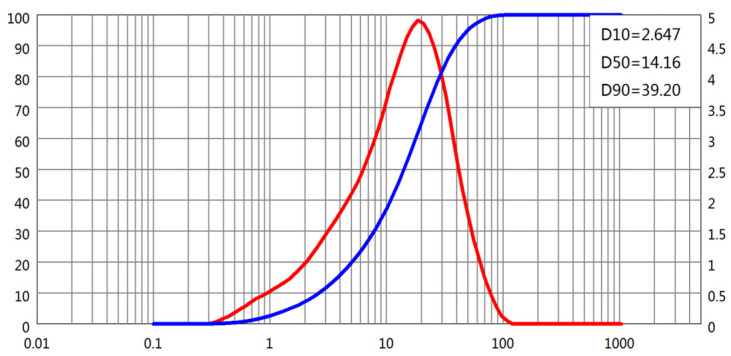
Particle size distribution of slag powder.

**Figure 4:**
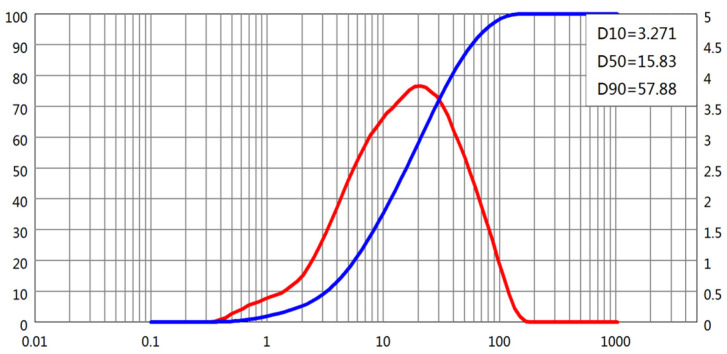
Particle size distribution of limestone powder.

The particle size distributions (PSDs) of all four powders were obtained using laser diffraction. D-values corresponding to 10%, 50%, and 90% cumulative passing (D10, D50, and D90, respectively) were extracted from the PSD curves, as shown in Figure 1, Figure 2, Figure 3 and Figure 4. The cement and slag powder exhibited relatively narrow distributions centered around 10–30 μm, while the fly ash showed a broader distribution with higher D90 values due to the presence of larger microspheres. The limestone powder had a slightly coarser profile, which could influence its packing behavior and interaction with admixtures.

These material characterizations provide the fundamental input parameters for modeling the rheological behavior of the paste system. In subsequent sections, these powder properties will be directly correlated with model parameters such as solid volume fraction, plastic viscosity, and yield stress.

### 2.2. Mix Design and Rheological Test Method

To systematically investigate the rheological behavior of cement pastes containing commonly used mineral admixtures, a factorial experimental matrix was developed to decouple the effects of water-to-binder ratio (w/b), mineral type, and dosage. Chemical admixtures were intentionally excluded in the initial phase of testing to avoid confounding effects caused by interactions between superplasticizers and powder surfaces, which are known to vary in charge density, hydration rate, and adsorption affinity [13,27,28]. This stepwise approach enables clearer interpretation of individual and combined factor effects on paste rheology.

Three types of supplementary cementitious materials (SCMs)—Class F fly ash (FA), S95-grade slag powder (SL), and ground limestone powder (ST)—were independently evaluated. Each was blended with cement at replacement levels of 9%, 18%, 27%, and 36% by mass. A control group without SCMs was included for baseline comparison. Five w/b ratios were selected (0.30, 0.35, 0.40, 0.45, 0.50), spanning the typical range for conventional and high-performance concrete. This matrix resulted in 65 unique mix designs. To ensure consistent test conditions and eliminate sample size effects on flow, each mixture was prepared at a fixed volume of 500 mL, as recommended in previous rheological protocols [29,30].

A second experimental set was designed to isolate the effect of chemical admixtures. Polycarboxylate ether (PCE)-based superplasticizers with varying water-reduction capacities were incorporated into pastes containing 20 vol.% FA. Superplasticizer dosage was varied incrementally to observe its influence on floc dispersion and water release behavior, which are known to strongly affect both yield stress and plastic viscosity [31,32].

**Figure 5:**
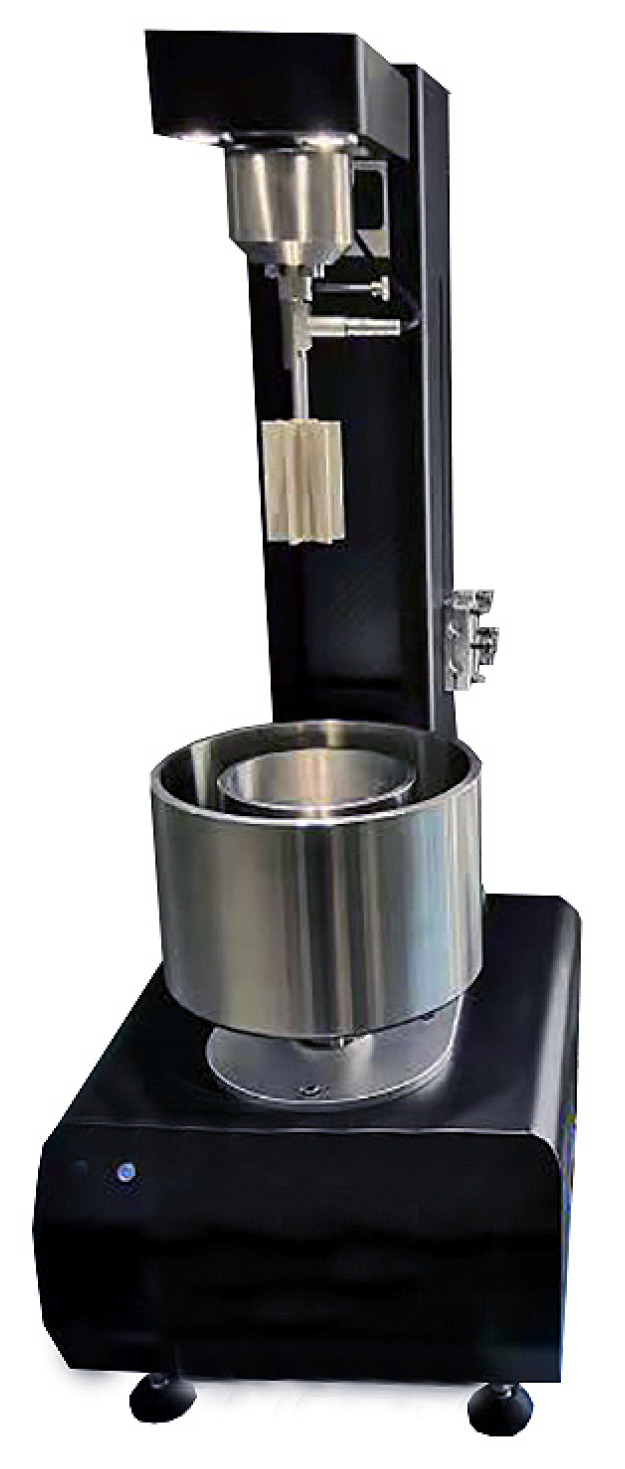
Mortar rheometer used for flow testing (LBY-II, Tianjin Gongyuan).

Rheological measurements were carried out using a rotational coaxial cylinder rheometer (Model LBY-II, Tianjin Gongyuan Instrument Co., Tianjin, China), shown in Figure 5. The device features a torque resolution of 0.01 N·mm, a shear rate range from 0.01 to 400 rpm, and an acquisition interval of 0.005–60 s. It accommodates samples with a maximum particle size of 3 mm and allows customized shear rate programming. Testing was conducted in a smooth cylindrical sample cup (110 mm height × 100 mm diameter), in accordance with protocols established by Roussel et al. [29] for cement paste rheology. Each test comprised a controlled shear ramp-up and ramp-down to capture both static yield stress and plastic viscosity, which were computed using the Bingham model.

This rigorous test design and controlled sample preparation ensured consistent and comparable rheological data across all mix compositions, thereby enabling model calibration based on measurable particle properties and mix parameters.

## 3. Experimental Results and Analysis

### 3.1. Effect of Solid Volume Fraction on Rheology

Portland cement without any mineral admixture or chemical admixtures. The compositions were designed to maintain a constant total volume of 500 mL for each sample, and the powder volume fraction (ϕ) was calculated based on the mass and density of cement and water. The detailed mix proportions and corresponding rheological properties are listed in Table 3.

**Figure 6:**
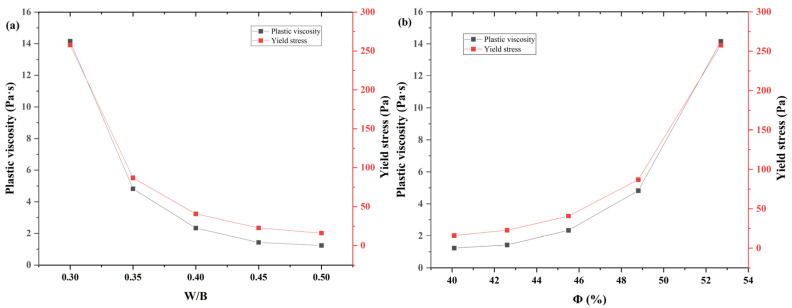
Effect of water–glue ratio on slurry fluidity. (**a**) W/B; (**b**) Solid phase volume percentage.

The plastic viscosity and yield stress decreased nonlinearly with increasing w/b ratio (i.e., decreasing solid volume fraction), as shown in Figure 6. This trend is consistent with classical suspension rheology, where higher particle concentrations increase interparticle interactions and hinder flow initiation [2,33].

**Figure 7:**
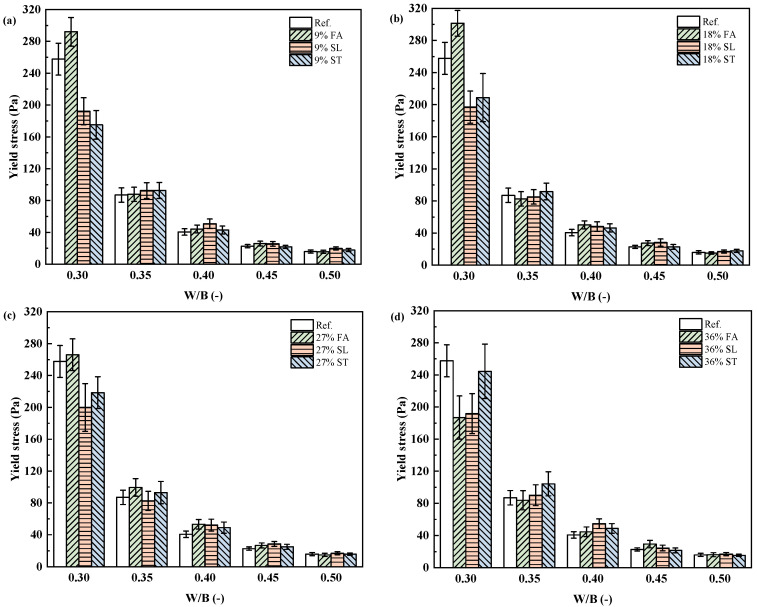
Yield stress variation with w/b ratio for different binder compositions. (**a**) 9% mineral admixture; (**b**) 18% mineral admixture; (**c**) 27% mineral admixture; (**d**) 36% mineral admixture.

**Figure 8:**
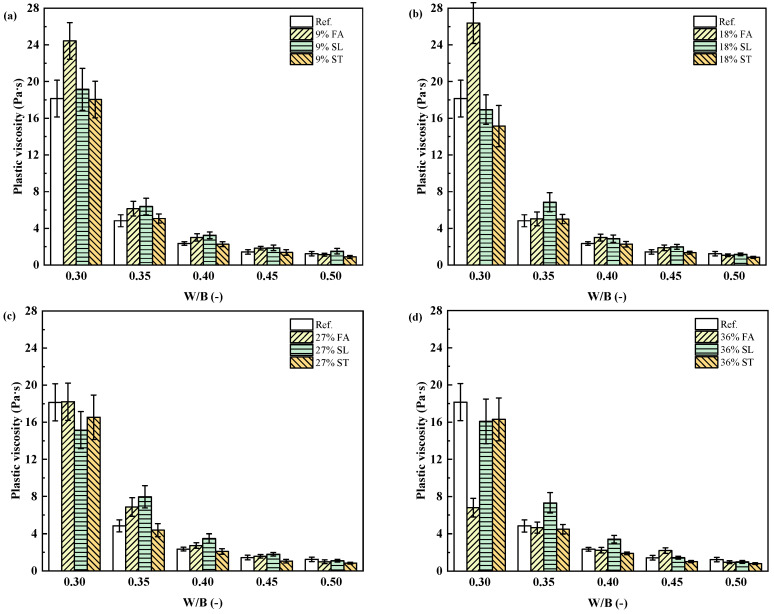
Plastic viscosity variation with w/b ratio for different binder compositions. (**a**) 9% mineral admixture; (**b**) 18% mineral admixture; (**c**) 27% mineral admixture; (**d**) 36% mineral admixture.

To further generalize this behavior, the rheological data from 65 mixtures with varying mineral admixture types and dosages were plotted against w/b ratio. As shown in Figure 7 and Figure 8, both plastic viscosity and yield stress generally decreased with increasing w/b. However, the effect of mineral admixtures became more pronounced at lower w/b ratios, especially for pastes containing finer or more reactive powders.

In modeling such behavior, solid volume fraction ϕ and the maximum packing density $\phi_{max}$ are recognized as key parameters. Krieger and Dougherty [21] developed a widely used model for concentrated suspensions:

(1) $$\;\mu \left(\phi \right)=\mu_{0}{\left(1-\frac{\phi }{\phi_{max}}\right)}^{-\left[\eta \right]\phi_{max}},$$

where $\mu_{0}$ is the plastic viscosity of the liquid phase, $\left[\eta \right]$ is the intrinsic viscosity determined by particle shape, and $\phi_{max}$ is the theoretical maximum packing fraction. In cement pastes, $\mu_{0}$ is typically taken as that of water, and $\left[\eta \right]$ ≈ 2.5–4 depending on flocculation.

For yield stress, Flatt [34] proposed a semi-empirical model based on interparticle spacing:

(2) $$\;\tau =m_{1}\frac{\phi^{3}}{\phi_{max}\left(\phi_{max}-\phi \right)},$$

where $m_{1}$ is an empirical parameter that accounts for electrochemical and surface interactions. These models rely on parameters that are difficult to measure directly in cement pastes due to hydration reactions, flocculation, and particle adsorption.

Notably, the effective solid volume fraction in cement paste is not merely the dry powder volume but includes absorbed and entrapped water within flocs [35]. The maximum packing fraction $\phi_{max}$ is also affected by particle shape, dispersion state, and admixtures [36,37,38]. Therefore, assuming constant $\phi_{max}$ (e.g., 0.64 or 0.74) can introduce large errors.

To simplify calibration, Liu et al. [39] proposed an alternative viscosity model:

(3) $$\;\mu \left(\phi \right)=\mu_{0}{\left[a_{f}\left(\phi_{max}-\phi \right)\right]}^{-n},$$

where $a_{f}$ is a parameter related to particle surface area and morphology, and *n* is a suspension-specific exponent, often taken as 2 for cementitious pastes.

In this study, model parameters were fitted using the five pure cement paste mixtures. The best-fit results were:

- $\phi_{max}$ = 57.9%,
- $a_{f}$ = 0.164,
- R^2^ = 0.999 (for viscosity model, Equation (3)).

For yield stress, fitting Equation (2) yielded:

- $\phi_{max}$ = 54.85%,
- $m_{1}$ = 21.03 Pa,
- R^2^ = 0.983.

**Figure 9:**
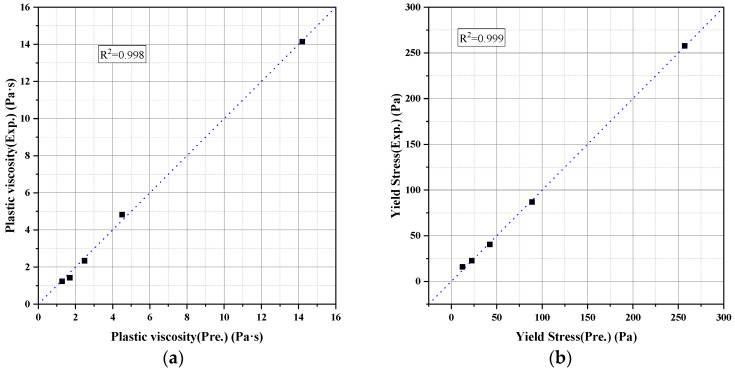
Model predictions versus experimental results for (**a**) plastic viscosity and (**b**) yield stress of cement paste.

Adjusted unified parameters are listed in Table 4, and predicted vs. measured results are plotted in Figure 9.

These results confirm that using only three fitted parameters—$\phi_{max}$, $m_{1}$, and $a_{f}$—the constitutive models can accurately capture the nonlinear dependence of paste rheology on solid concentration, offering a practical basis for predictive rheology in cementitious systems.

### 3.2. Effect of Mineral Admixtures on Paste Rheology

To systematically investigate the influence of mineral admixtures on paste rheology, the solid phase was defined solely as the volume of fine powders (cement and mineral admixtures), excluding entrained water and flocculated water volumes. Based on the previously established models for plastic viscosity and yield stress—Equations (2) and (3)—the impact of mineral admixtures was reduced to variations in three key fitting parameters: the maximum packing fraction $\phi_{max}$, the empirical yield stress coefficient $m_{1}$, and the flow resistance factor $a_{f}$. This simplified framework enables more efficient prediction and interpretation of flow behavior across different mineral compositions.

**Figure 10:**
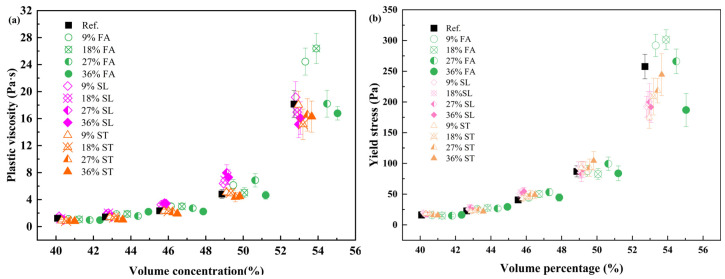
(**a**) Plastic viscosity and (**b**) yield stress of pastes with varying powder volume concentrations and mineral admixture types.

Since the water-to-binder ratio (w/b) is traditionally defined on a mass basis, direct comparisons of rheological behavior between mixtures containing different powders require adjustment for density differences. Accordingly, the powder volume concentration ϕ was recalculated based on the actual densities of the binders and water, allowing for consistent evaluation across compositions. The resulting trend of plastic viscosity versus ϕ is shown in Figure 10.

The results reveal that while plastic viscosity increases with solid volume fraction for all mixes, the rate of increase is strongly influenced by the type of mineral admixture. The differences become particularly significant at higher concentrations, reflecting the impact of powder characteristics such as particle size distribution, surface area, and reactivity. Fly ash, slag, and limestone powder each exhibit distinct rheological signatures attributable to these properties [13,40,41].

In the absence of superplasticizers, both yield stress and viscosity increased with powder content, following the exponential trends described by Equations (2) and (3). These results validate the approach of using three tunable parameters—$\phi_{max}$, $m_{1}$, and $a_{f}$—to characterize the flow resistance of different paste systems.

To quantify the effect of mineral admixture type and dosage, 65 paste formulations were analyzed using a three-parameter nonlinear regression method. The fitted values for each mix are summarized in Table 5.

The results demonstrate that the influence of mineral admixtures on rheological properties can be captured through shifts in the model parameters. For example, fly ash increases the maximum packing fraction $\phi_{max}$ and reduces $m_{1}$, reflecting enhanced packing and lower interparticle friction due to its spherical morphology [42]. Conversely, limestone powder has a relatively high $m_{1}$, indicating stronger interparticle interactions and limited dispersion.

**Figure 11:**
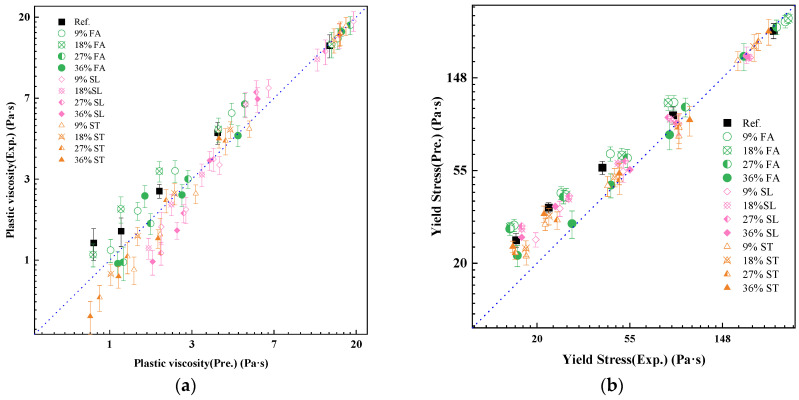
Comparison of predicted and measured values for (**a**) plastic viscosity and (**b**) yield stress in pastes with mineral admixtures.

By integrating the fitted parameters into the rheological models, the predicted plastic viscosity and yield stress were compared with experimental results for all 65 mixtures. As shown in Figure 11, the predicted values align closely with the measured data, with R^2^ values exceeding 0.85 for both rheological indices.

These results confirm that the proposed three-parameter framework provides a robust basis for capturing the rheological behavior of blended pastes, offering both physical interpretability and predictive capability. It also provides a means to incorporate powder characteristics (e.g., fineness, morphology) into a model-driven mix design.

### 3.3. Effect of Superplasticizer on Paste Rheology

The previously developed constitutive models (Equations (2) and (3)) describe the yield stress and plastic viscosity of cementitious pastes as functions of powder volume concentration and a set of material-specific parameters, including the maximum packing fraction $\phi_{max}$. In earlier sections, the influence of mineral admixtures was modeled by fitting three parameters: $\phi_{max}$, $a_{f}$, and $m_{1}$, which collectively represent the packing behavior, interparticle interactions, and flow resistance.

When polycarboxylate ether (PCE)-based superplasticizers are introduced, their primary effect is to disperse flocculated particles, thereby releasing entrained water and increasing the effective paste fluidity. To integrate this mechanism into the rheological model, we assume that the superplasticizer modifies only $\phi_{max}$, while the other two parameters, $a_{f}$ and $m_{1}$, remain constant for a fixed powder composition. This assumption allows the effect of the superplasticizer to be captured by a single variable—its influence on the virtual maximum packing fraction.

The water-reducing efficiency of the superplasticizer is expressed as a water-reduction ratio, defined as the percentage decrease in water required to achieve a given level of flowability. This metric enables consistent quantification of the dispersion effect across different admixture dosages.

To evaluate the validity of this approach, 16 paste mixtures were prepared using ordinary Portland cement and 20 vol.% fly ash, with a PCE superplasticizer introduced at varying dosages (0.4–1.0% by binder weight). The paste compositions and rheological properties are summarized in Table 6. Water-to-binder ratios and powder concentrations were adjusted to maintain a fixed powder composition while varying the superplasticizer dosage.

**Figure 12:**
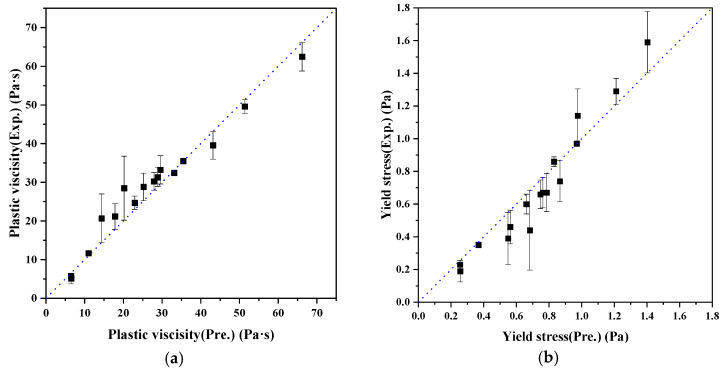
Relationship between PCE dosage and fitted maximum packing fraction $\phi_{max}$ in paste model. (**a**) Plastic viscosity; (**b**) Yield stress.

Fitting the model to these 16 data points using a nonlinear regression method, the parameters $a_{f}$ and $m_{1}$ were held constant at 0.034 and 0.72, respectively, while $\phi_{max}$ was treated as a function of the superplasticizer dosage. The fitted results, shown in Figure 12, indicate a clear trend: $\phi_{max}$ increases with SP dosage, reflecting the enhanced dispersion and packing efficiency achieved through floc breakdown.

Based on these results, the effect of superplasticizer can be incorporated into the constitutive equations by substituting $\phi_{max}\;$= $\;\phi_{max}$(SP), allowing Equations (4) and (5) to remain structurally unchanged while accounting for admixture action:

(4) $$\;\tau =m_{1}\frac{\phi^{3}}{\phi_{max}(SP)\left(\phi_{max}(SP)-\phi \right)},$$

(5) $$\mu \left(\phi \right)=\mu_{0}{\left[a_{f}\left(\phi_{max}(SP)-\phi \right)\right]}^{-n}.$$

These updated models preserve the physical interpretability of each parameter and offer a practical pathway to predict paste rheology under variable superplasticizer dosages. The high R^2^ values (typically > 0.90 for both τ and η) validate the model’s predictive performance.

This method provides a simplified yet robust framework for incorporating admixture dosage into rheological modeling, avoiding the need to re-estimate all parameters while maintaining high accuracy. Moreover, it supports the broader use of this model in intelligent mix design systems where variable superplasticizer demand must be balanced with fluidity and stability constraints.

## 4. Conclusions

This study proposed a constitutive rheological model for cementitious pastes based on solid volume fraction and powder characteristics and systematically evaluated the effects of water-to-binder ratio, mineral admixtures, and superplasticizer dosage on paste rheology. Traditional rheological models, such as Bingham, Herschel–Bulkley, and especially Krieger–Dougherty, struggle to accurately predict cement paste behavior because key parameters—like effective solid volume fraction and maximum packing density—are inherently difficult to measure directly in such complex, chemically reactive systems due to evolving flocculation and entrapped water. Our model explicitly mitigates these direct measurement challenges. Unlike purely empirical approaches, our model offers a novel theoretical framework rooted in the fundamental properties of the powder constituents and their interactions. It explicitly addresses the limitations of classical models by introducing a physically interpretable “virtual maximum packing fraction” and directly linking macroscopic rheology to readily measurable material characteristics, thus providing a more robust and predictive tool. The major conclusions are as follows:

1. Solid volume fraction is the dominant factor governing paste rheology.
  Both plastic viscosity and yield stress increase exponentially as the powder volume fraction approaches the maximum packing limit. A strong nonlinear dependence was observed and accurately captured using modified suspension models. Model fitting for plain cement paste yielded high predictive accuracy (R^2^ > 0.98) for both viscosity and yield stress, demonstrating a level of predictive capability beyond simple curve-fitting approaches.
2. The influence of mineral admixtures on flowability can be fully quantified through three fitted parameters.
  The rheological impact of fly ash, slag, and limestone powder was successfully modeled by changes in the maximum packing fraction $\phi_{max}$, the yield stress coefficient $m_{1}$, and the flow resistance factor $a_{f}$. Fly ash significantly enhanced flowability by increasing $\phi_{max}$ and reducing interparticle interactions, while slag and limestone exhibited milder effects. All 65 paste formulations were well predicted with R^2^ > 0.85, highlighting our model’s ability to quantitatively describe the complex interactions of SCMs, a feature typically not addressed by generalized rheological models.
3. Superplasticizer action can be simplified as a modulation of virtual packing density.
  The polycarboxylate-based superplasticizer increased the effective $\phi_{max}$ by dispersing flocs and releasing entrapped water. By fixing $a_{f}$ and $m_{1}$, the variation in $\phi_{max}$ with dosage allowed the accurate prediction of flow parameters across 16 tested formulations, confirming the model’s robustness in admixture-modified systems. This simplification provides a physically intuitive mechanism for superplasticizer effects, which is a significant advancement over models that treat admixture effects as arbitrary fitting parameters, and offers a pathway to predict their impact without complex direct rheological measurements for every formulation.
4. A unified, parameter-efficient model framework was established.
  Through minimal parameterization, the model bridges raw material characteristics and macroscopic rheological behavior. It enables forward prediction of plastic viscosity and yield stress based on volume concentration and material properties, with clear physical interpretation and practical adaptability. This contrasts sharply with many existing models that rely heavily on empirically derived constants or require extensive, material-specific calibration, making our framework more universally applicable for diverse cementitious systems. By focusing on easily measurable input parameters and the derived $\phi_{max}$, our model bypasses the practical difficulties associated with direct measurement of effective solid volume fractions in real-time, offering a truly predictive and actionable tool.

The proposed model offers a predictive tool for paste rheology optimization in concrete mix design. It supports performance-driven proportioning of high-performance concrete, self-consolidating concrete, and 3D printable mixtures, particularly when integrated with digital design platforms or AI-assisted formulation systems.

## Figures and Tables

**Table 1:** Physical properties of cement.

Blaine (m^2^/kg)	Density (kg/m^3^)	Standard Consistency (%)	Initial Set (min)	Final Set (min)	Flexural Strength (MPa)	Compressive Strength (MPa)
317	2990	28.4	193	266	9.3 (28 d)	43.2 (28 d)

**Table 2:** Physical characteristics of SCMs and cement.

Material	Density (g/cm^3^)	Blaine Fineness (m^2^/kg)	Surface Area per Unit Volume (m^2^/m^3^)
Cement (C)	2.99	315.2	0.94
Fly ash (FA)	2.35	453.4	1.07
Slag powder (SL)	2.88	343.6	0.99
Limestone powder (ST)	2.7	311.2	0.84

**Table 3:** Rheological properties of cement pastes at different w/b ratios.

Mix	Cement (g)	Water (g)	w/b	ϕ (%)	Plastic Viscosity (Pa·s)	Yield Stress (Pa)
1	794	238	0.3	52.7	14.15	25.8
2	735	257	0.35	48.8	4.82	8.7
3	684	273	0.4	45.5	2.34	4.1
4	639	288	0.45	42.6	1.43	2.3
5	600	300	0.5	40.1	1.23	1.6

**Table 4:** Calibrated model parameters for pure cement paste.

$\phi_{max}$	$m_{1}$ (Pa)	$a_{f}$	R^2^ (μ)	R^2^ (τ)
57.49%	40.35	0.155	0.962	0.94

**Table 5:** Fitted rheological parameters for cement pastes containing mineral admixtures.

Admixture Type	Replacement	$\phi_{max}$ (%)	$m_{1}$ (Pa)	$a_{f}$	R^2^ (μ)	R^2^ (τ)
FA	9%	58	38.4	0.156	0.98	0.87
FA	18%	58.3	37	0.157	0.86	0.88
FA	27%	58.8	35.4	0.158	0.98	0.95
FA	36%	59.3	34	0.159	0.95	0.9
SL	9%	57.8	39.5	0.155	0.86	0.99
SL	18%	58	39.1	0.155	0.86	0.99
SL	27%	58.3	38.4	0.155	0.86	0.98
SL	36%	58.4	38	0.155	0.86	0.99
ST	9%	57.6	40.1	0.156	0.99	0.86
ST	18%	57.8	39.9	0.157	0.98	0.98
ST	27%	58	39.6	0.158	0.99	0.98
ST	36%	58.2	39.3	0.159	0.98	0.99

**Table 6:** Mix proportions and rheological properties of pastes with PCE superplasticizer.

Mix	Vp (L)	Vw (L)	ϕ (%)	SP (%)	Cement (kg/m^3^)	FA (kg/m^3^)	Water (kg/m^3^)	SP (kg/m^3^)	Yield Stress τ (Pa)	Plastic Viscosity η (Pa·s)
1	459	533	45.80%	0.80%	1130	234	533	10.91	0.35	11.66
2	459	531	45.90%	0.90%	1131	234	531	12.29	0.23	5.77
3	460	530	45.90%	1.00%	1132	234	530	13.67	0.19	5.11
4	503	493	50.20%	0.40%	1290	256	493	6.19	1.95	62.48
5	503	490	50.30%	0.50%	1293	257	490	8.52	0.97	35.48
6	504	489	50.30%	0.60%	1293	257	489	8.83	0.74	33.22
7	504	489	50.40%	0.60%	1294	257	489	9.3	0.67	28.8
8	478	517	47.80%	0.40%	1229	244	517	5.89	1.14	39.57
9	479	515	47.90%	0.50%	1230	244	515	7.37	0.66	30.23
10	503	491	50.30%	0.50%	1292	257	491	7.74	1.29	49.6
11	504	487	50.40%	0.70%	1295	257	487	10.87	0.39	20.68
12	479	516	47.90%	0.40%	1229	244	516	6.63	0.86	32.42
13	479	516	47.90%	0.50%	1230	244	516	7.08	0.67	31.31
14	504	488	50.40%	0.60%	1294	257	488	10.08	0.44	28.48
15	479	514	47.90%	0.50%	1231	244	514	8.11	0.6	24.68
16	480	513	47.90%	0.60%	1232	245	513	8.86	0.46	21.16

## Data Availability

The original contributions presented in this study are included in the article. Further inquiries can be directed to the corresponding author.
